# Pseudomyxoma Peritonei Presenting With Progressive Abdominal Distension and Abdominal Hernia: A Case Report

**DOI:** 10.7759/cureus.104402

**Published:** 2026-02-27

**Authors:** Menna M Marouf, Sarah Hady, Batool Musleh, Khalid E Attia, Ibrahim El Nogoomi

**Affiliations:** 1 Clinical Sciences, College of Medicine, University of Sharjah, Sharjah, ARE; 2 Radiology, Kuwait Hospital, Sharjah, ARE; 3 General Surgery, Kuwait Hospital, Sharjah, ARE

**Keywords:** abdominal distention, appendiceal mucinous neoplasm, cytoreductive surgery, hyperthermic intraperitoneal chemotherapy, pseudomyxoma peritonei

## Abstract

Pseudomyxoma peritonei (PMP) is a rare clinical condition characterized by progressive mucinous ascites, most commonly arising from a low-grade appendiceal mucinous neoplasm (LAMN). We report the case of a 53-year-old man with a past medical history of type 2 diabetes mellitus who presented with progressive abdominal distension, constipation, weight loss, and a supraumbilical hernia. Imaging modalities, including ultrasonography, magnetic resonance imaging (MRI), and computed tomography (CT), revealed omental caking, ascites, and hepatic scalloping. Diagnostic laparoscopy revealed mucinous nodules and an abnormal appendiceal tip, and histopathology confirmed LAMN with PMP. The patient had an uneventful recovery following laparoscopic appendectomy and peritoneal biopsies and was referred to a specialized oncology center for consideration of cytoreductive surgery and hyperthermic intraperitoneal chemotherapy (HIPEC). This case highlights the diagnostic challenges of PMP and underscores the importance of early detection and multidisciplinary management to achieve optimal outcomes.

## Introduction

Pseudomyxoma peritonei (PMP) is a rare and slowly progressing condition characterized by the accumulation of mucinous ascites and neoplastic mucin-producing epithelial cells within the peritoneal cavity. These cells disseminate across the peritoneal surfaces and continue to secrete mucin. Over time, this leads to a progressive increase in intra-abdominal volume and pressure, causing the abdomen to swell and eventually compress nearby organs [1,2]. Clinically, patients can present with abdominal distension and increased abdominal girth, changes in bowel habits, loss of appetite and weight, and new-onset hernias. In many cases, the diagnosis is made incidentally during surgery for what is presumed to be another condition. These features, especially when accompanied by ascites, can be mistakenly attributed to other conditions such as chronic liver disease, delaying the correct diagnosis.

Radiological findings such as omental caking, hepatic scalloping, and septated ascites on CT or MRI are highly suggestive of PMP; however, direct visualization and histopathological analysis play a central role in confirming the diagnosis of the disease. Many cases of PMP are diagnosed incidentally during surgical exploration for other medical concerns due to the condition's nonspecific and vague presentation, which can delay recognition [1].

The appendix is the most common primary site of origin for low-grade appendiceal mucinous neoplasm (LAMN); however, other mucinous tumors originating from the ovary, colon, or urachus can also be sources [3-6]. In many cases involving women, the ovaries are affected secondarily by metastasis from an appendiceal primary [4,5].

PMP is rare, with an estimated incidence of one to two cases per million individuals per year [7]. The prognosis of PMP depends on both tumor grade and the extent of peritoneal spread. Without treatment, PMP is progressive and potentially fatal due to complications such as bowel obstruction, malnutrition, and infection [8]. However, hyperthermic intraperitoneal chemotherapy (HIPEC) in conjunction with cytoreductive surgery (CRS) has become the gold standard of care, providing notable survival advantages, particularly for patients with low-grade disease. The prognosis of PMP is variable and largely determined by tumor histology and grade, as well as the combination of complete cytoreduction and HIPEC [9-11].

## Case presentation

A 53-year-old Yemeni male with a known history of type 2 diabetes mellitus, managed with oral hypoglycemic medications, was referred to the surgical clinic for evaluation of a supraumbilical hernia and persistent abdominal pain over the past several months. He also reported chronic constipation and unintentional weight loss of around 10 kilograms from a baseline weight of 87 kilograms, last measured approximately one year prior to presentation. He denied anorexia, nausea, vomiting, fever, or night sweats. He denied smoking, alcohol consumption, or illicit drug use and had no history of previous surgeries. He had been reasonably well before the onset of his abdominal pain and was managing his diabetes with oral medications without significant issues.

Upon general examination, the patient was afebrile and vitally stable. He appeared generally well, with no conjunctival pallor, jaundice, scleral icterus, lymphadenopathy, or peripheral edema. Abdominal examination revealed a soft abdomen with positive shifting dullness suggestive of ascites, a supraumbilical hernia, and divarication of the recti. There was no localized tenderness or organomegaly.

Initial laboratory workup revealed that tumor markers, including carbohydrate antigen (CA) 19-9 and alpha-fetoprotein (AFP), were within the normal range; however, carcinoembryonic antigen (CEA) levels were elevated at 10.2-18.0 ng/mL (reference range below 3.8 ng/mL).

**Figure 1:**
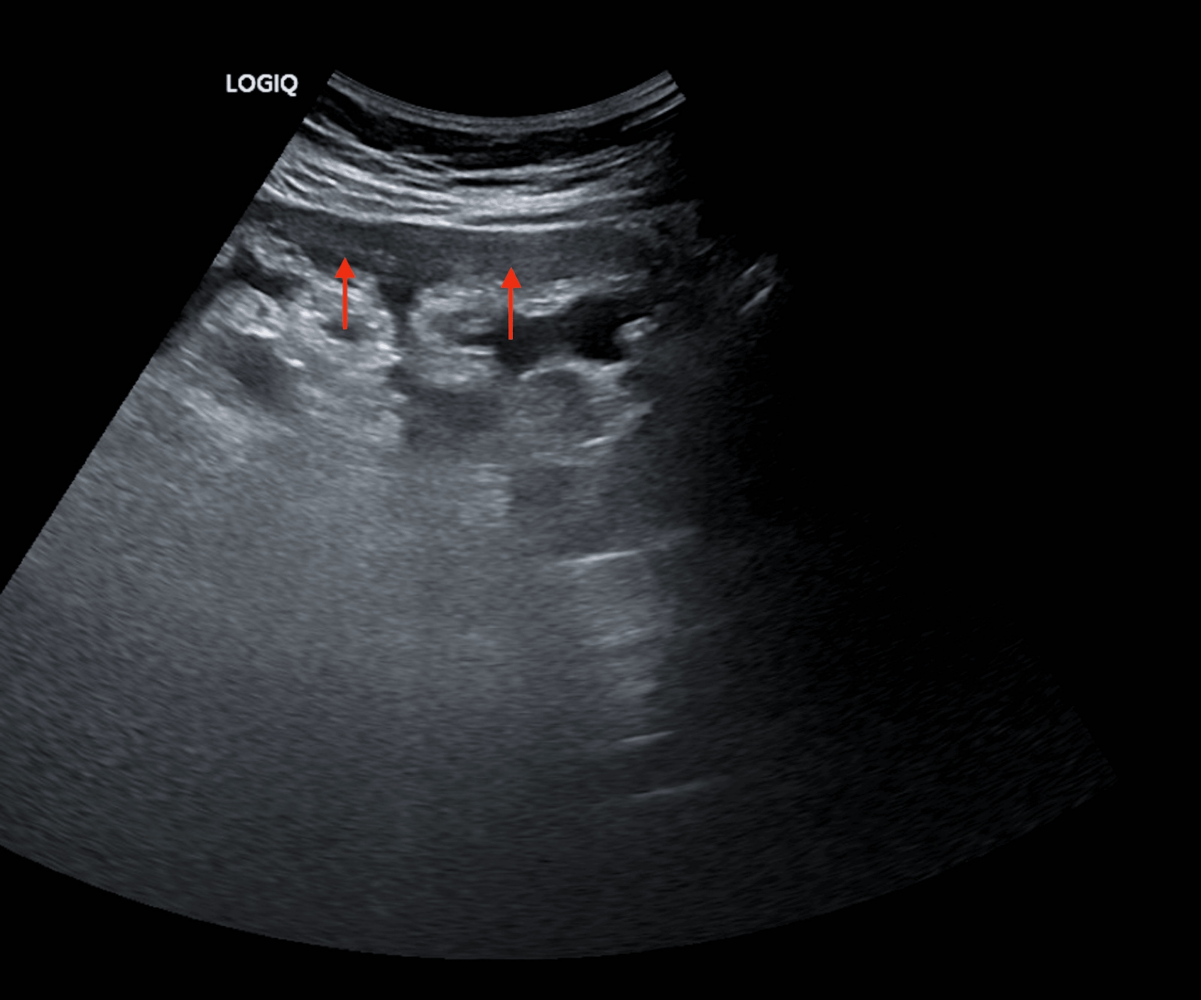
Abdominal ultrasonography showing omental thickening Ultrasound image showing omental thickening, or omental 'caking', indicated by the red arrows, which is suggestive of pathologic involvement of the omentum.

Abdominal ultrasonography revealed mild ascites and omental thickening with peritoneal deposits (Figure 1). Upper gastrointestinal endoscopy and colonoscopy were unremarkable. Contrast-enhanced computed tomography (CECT) of the abdomen revealed omental caking, mild ascites, an irregular liver outline suggestive of early cirrhosis, four small paraumbilical hernias (5 mm each), and a left renal calculus.

**Figure 2:**
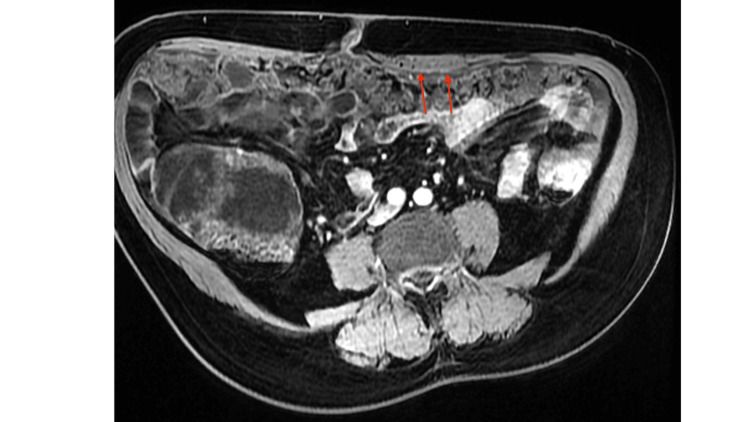
Axial MRI in lava sequence, showing omentum thickening Thickening denoted by the red arrows, suggestive of infiltration of the omental fat.

Further evaluation with magnetic resonance imaging (MRI) of the abdomen demonstrated omental caking with diffusion restriction, suggestive of either peritoneal carcinomatosis or granulomatous peritonitis (Figure 2). Other findings included septated chronic peritoneal fluid collections, hepatic scalloping, which is the most characteristic radiological sign of PMP, early chronic liver disease, and mild ascites.

Based on the clinical features and imaging findings, the following differentials were considered: PMP, peritoneal carcinomatosis, tuberculous peritonitis, and peritoneal mesothelioma, all of which can present with similar clinical features and overlapping imaging findings, including omental thickening, ascites, and peritoneal deposits, necessitating tissue biopsy and histopathological analysis.

**Figure 3:**
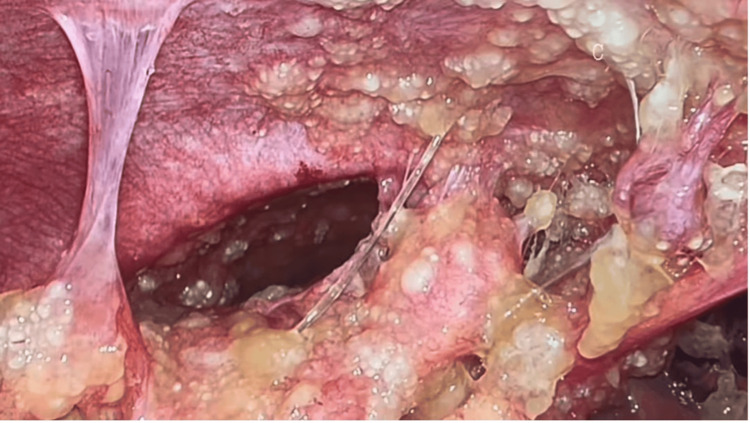
Omental caking observed on diagnostic laparoscopy Multiple lesions are seen adherent to the abdominal wall.

**Figure 4:**
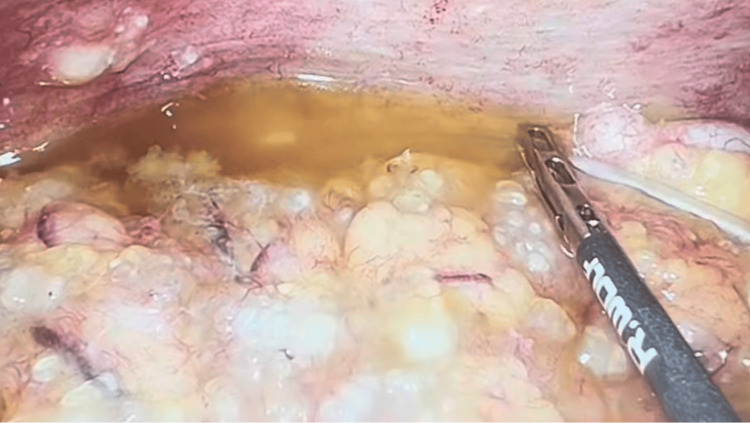
Aspiration of the mucinous ascitic fluid.

To establish a definitive diagnosis, the patient underwent diagnostic laparoscopy with peritoneal biopsies, appendectomy, and aspiration of ascitic fluid. Intraoperatively, mucinous ascites was noted in the peritoneal cavity, with multiple peritoneal nodules scattered across the peritoneal surfaces and extensive omental caking (Figure 3). The appendiceal tip appeared abnormal and nodular. Peritoneal fluid was aspirated and sent for analysis, and biopsies were obtained from the omentum and peritoneal nodules (Figure 4).

Histopathological examination of the resected appendix confirmed an LAMN, classified as grade I according to the WHO classification. The peritoneal and omental biopsies demonstrated low-grade mucinous neoplasia consistent with PMP.

**Figure 5:**
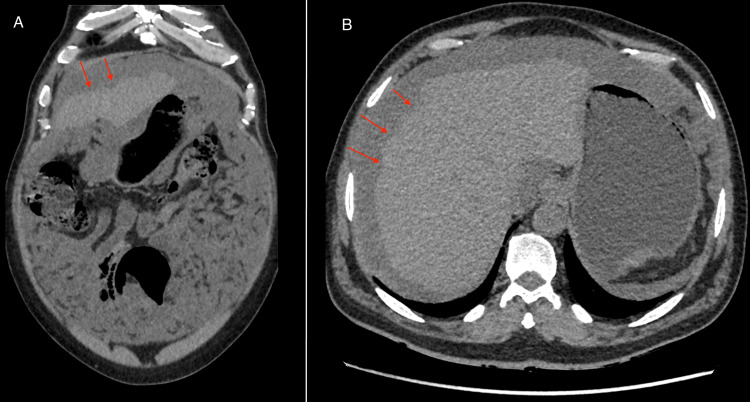
(A) Axial CT scan of the abdomen without contrast, showing hepatic scalloping and mild ascites. (B) Coronal view of the CT scan showing scalloping of the liver. Red arrows in figures A and B indicate the hepatic scalloping, caused by the thick mucinous substances creating indentations on the surface of the liver.

Following histopathological confirmation, he was referred to a specialized oncology center for treatment with CRS and HIPEC. At the time of referral, he was clinically stable, tolerating diet, and his surgical wounds were healing well. Subsequent outpatient clinic visits were uneventful. He presented again to the clinic several weeks later with complaints of left renal colic. A CT KUB was obtained, which revealed persistent abdominal ascites, omental thickening, and scalloping of the liver (Figure 5).

## Discussion

PMP is characterized by the accumulation of mucinous material in the peritoneal cavity, giving it its unique nickname of “jelly belly” [1,12]. The diagnostic challenges associated with PMP arise from its vague, nonspecific, and diverse clinical presentation in patients. While PMP commonly presents with vague symptoms such as abdominal pain, distension, nausea, and changes in bowel habits [3,13], our patient’s case was notable for a concurrent supraumbilical hernia, a less frequent but recognized presentation [14], likely resulting from the chronic increase in intra-abdominal pressure caused by the accumulating mucinous material. This demonstrates the uniqueness of PMP in its diverse presentation.

The incidence of PMP is higher in females than in males, with a 11:9 ratio and a mean age at onset of 53 years [3]. In males, it is most commonly associated with a ruptured mucinous lesion of the appendix. Less commonly, it may arise from the colorectum, gallbladder, pancreas, urachus, urinary bladder, breast, or lung; however, these occurrences are typically rare [3,4]. In females, the ovaries are frequently involved, often secondarily to an appendiceal primary, which can sometimes make it challenging to distinguish the original site [4,5,13].

Imaging plays a considerable role as a diagnostic modality. Previous studies have highlighted the role of CECT in the diagnosis of PMP [12]. CECT allows differentiation between mucinous substances and simple fluid based on differences in attenuation, with mucinous substances demonstrating areas of low attenuation mixed with areas of higher attenuation from solid tumor elements. This is accompanied by “scalloping of visceral surfaces,” due to the thick mucinous substance creating indentations on the surfaces of the liver and spleen [4,12,15]. Similarly, MRI demonstrates low attenuation, scalloping of visceral surfaces, and the presence of peritoneal implants that may exert pressure on the bowel loops [4].

The initial imaging finding of an irregular liver outline raised the possibility of early chronic liver disease, which in this patient with diabetes could potentially be attributed to nonalcoholic fatty liver disease (NAFLD). However, hepatic scalloping and peritoneal deposits were more consistent with PMP on further evaluation, highlighting the importance of not attributing ascites and hepatic irregularities solely to liver disease without considering other peritoneal pathology.

Tumor markers play a central role in the diagnosis, prognosis, and postoperative follow-up of PMP, including CA-125 and particularly CEA. In our case, CEA was elevated, approximately three to five times the upper limit of normal, and histopathological examination confirmed low-grade mucinous neoplasia, which is consistent with published literature indicating that low-grade PMP is associated with lower CEA levels compared with high-grade disease, in which elevations are more pronounced [12]. CA-125 in particular is associated with PMP of ovarian origin [12].

Biopsy also plays a central role in the diagnosis and grading of the disease, which classifies PMP into low- or high-grade forms. Histologically, PMP has been classified into two forms: low-grade mucinous carcinoma peritonei, formerly called disseminated peritoneal adenomucinosis (DPAM), and high-grade mucinous carcinoma peritonei, formerly called peritoneal mucinous carcinomatosis (PMCA), with the former typically associated with a better prognosis, especially following complete CRS [6]. The cornerstone of management in PMP is CRS plus HIPEC. In some studies, following complete cytoreduction plus HIPEC, combined CRS and HIPEC demonstrated five-year survival rates ranging from 62% to 80% [9-11].

## Conclusions

PMP is a rare yet clinically significant condition that often presents insidiously with nonspecific and diverse complaints, such as abdominal distension, abdominal pain, diarrhea, weight loss, nausea and vomiting, and rarely, abdominal wall hernias. This case emphasizes the importance of maintaining a high index of suspicion in patients with unexplained clinical features, such as ascites, increased abdominal girth, and abdominal wall hernias. Diagnosis is further supported by radiological findings on CECT and MRI, such as omental caking, scalloping of visceral organs, and differences in attenuation that differentiate normal fluid from mucinous fluid. However, definitive diagnosis relies on direct visualization and histopathological confirmation, which is often discovered incidentally. CRS and HIPEC have been associated with improved survival outcomes in the literature; however, early recognition remains fundamental. Recognizing subtle, unexplained clinical signs in patients, along with the radiological features of PMP, enables timely referral to specialized centers, which offer optimal management and the best chance for survival, with survival rates exceeding 80%.
